# Longitudinal patterns in the skin microbiome of wild, individually marked frogs from the Sierra Nevada, California

**DOI:** 10.1038/s43705-021-00047-7

**Published:** 2021-09-01

**Authors:** Silas Ellison, Roland Knapp, Vance Vredenburg

**Affiliations:** 1 Department of Biology, San Francisco State University, San Francisco, California USA; 2 Sierra Nevada Aquatic Research Laboratory, University of California, Mammoth Lakes, CA USA; 3 Museum of Vertebrate Zoology, University of California, Berkeley, California USA

**Keywords:** Microbiome, Microbial ecology, Conservation biology

## Abstract

The amphibian skin microbiome has been the focus of numerous studies because of the protective effects that some bacteria provide against the pathogenic fungus *Batrachochytrium dendrobatidis*, which has caused a global panzootic among amphibians. However, the mechanisms driving community structure and function in the amphibian skin microbiome are still poorly understood, and longitudinal analyses of the skin microbiome have not yet been conducted in wild populations. In this study, we investigate longitudinal patterns in the skin microbiome of 19 individually marked adult frogs from two wild populations of the endangered Sierra Nevada yellow-legged frog (*Rana sierrae*), sampled over the course of 2 years. We found that individuals with low bacterial diversity (dominated by order Burkhorderiales) had significantly more stable bacterial communities than those with higher diversity. Amplicon sequence variants (ASVs) with high relative abundance were significantly less transient than those with low relative abundance, and ASVs with intermediate-level relative abundances experienced the greatest volatility over time. Based on these results, we suggest that efforts to develop probiotic treatments to combat *B. dendrobatidis* should focus on bacteria that are found at high relative abundances in some members of a population, as these strains are more likely to persist and remain stable in the long term.

## Introduction

The fungal pathogen *Batrachochytrium dendrobatidis* has caused the severe decline or extinction of over 200 amphibian species [1, 2]. This microscopic fungus infects the skin of amphibians and causes death in susceptible individuals by disrupting the skin electrolyte transport system [3, 4]. Studies over the past decade have identified hundreds of members of the amphibian skin bacterial microbiome that can inhibit the growth of *B. dendrobatidis*, both in vitro [5, 6] and in vivo [7, 8], and such studies have given rise to hope that targeted bioaugmentation interventions using *B. dendrobatidis*-inhibitory bacteria could help protect vulnerable populations of amphibians from epizootic outbreaks of *B. dendrobatidis* [9]. However, attempts to implement bioaugmentations in wild populations have often been hampered by limited persistence of target bacterial strains over time [7, 10, 11], which highlights the importance of understanding the fundamental community ecology of skin microbial communities, in particular how they fluctuate through time under natural conditions. Our knowledge of such temporal dynamics is currently limited, especially in the skin microbiome of animal species in wild populations. Here we report the results of a longitudinal study focusing on the skin microbiome of individually marked Sierra Nevada yellow-legged frogs (*Rana sierrae*) in two wild populations.

A number of important questions regarding temporal dynamics in the amphibian skin microbiome remain unanswered. For example, to what extent do community structure (measures incorporating microbial relative abundance) and membership (ignoring relative abundance) vary through time among amphibian hosts, and what other community characteristics (e.g., richness, evenness, dominant taxa) might be correlated with community variability? What behavior of bacterial amplicon sequence variants (ASVs) drive differences in community variability between individuals? To what extent do community functions, such as defense provided against fungal pathogens (e.g., *B. dendrobatidis*), fluctuate through time? Answering these questions requires a longitudinal study design, and such investigations are critical to gaining insight into the behavior of healthy and impaired microbial communities in amphibians and other animal hosts. For instance, a microbiome with excessive stability or variability might mask or accentuate natural seasonal or ontogenetic variation, potentially representing a dysbiotic state [12]. Temporal variability is also highly relevant to the development of treatments to mitigate diseased states: a highly variable bacterial community may be easier to manipulate experimentally, given a higher level of natural turnover in ASV presence and abundance, whereas a successfully introduced probiotic may be more likely to persist through time in a more stable bacterial community, given the lower levels of temporal variation.

In humans, a recent study found that skin microbial community structure was largely stable through time, although the skin microbiome of some individuals was more stable than others [13]. Individuals with a more stable microbiome tended to host microbial communities with less overall diversity; individuals with more variable communities tended to have higher diversity. A laboratory experiment examining temporal patterns and disease dynamics in the skin microbiome of the European common frog similarly found that bacterial community structure was less stable over time in frogs with higher microbiome diversity, but that stability of the 100 most abundant community members did not differ significantly between high-diversity and low-diversity individuals [14]. Two other recent studies have investigated temporal stability in the skin microbiome of individual newts in semi-natural settings (mesocosms or field enclosures): one study found that the skin microbial community structure of Eastern red-spotted newts was relatively stable across seasons and years, but that environmental disturbance significantly impacted community structure, richness, evenness, and phylogenetic diversity [15]. Another study found that community structure varied across the terrestrial-aquatic transition in three newt species, but that the proportion of bacteria predicted to inhibit the growth of *B. dendrobatidis* remained stable through time [16]. Other studies with repeated sampling of populations over time have found that bacterial community structure differed significantly between study years [17], seasons [18, 19], or based on environmental conditions, such as rainfall or temperature [20, 21].

Our study species, *R. sierrae*, lives at mid to high elevations in the Sierra Nevada mountains of California and the species has been extirpated from over 90% of its historical range [22]. Severe population declines in the early twentieth century, caused by the introduction of non-native fish, were followed by *B. dendrobatidis* epizootics (epidemic outbreaks in wildlife) beginning in the 1970s [23, 24]. Surviving populations typically display enzootic disease dynamics: recently metamorphosed juveniles experience high *B. dendrobatidis* infection intensities and mortality, whereas low-intensity *B. dendrobatidis* infections are highly prevalent among surviving adults [25]. However, a recent long-term study of *R. sierrae* population dynamics demonstrated that after many decades of significant declines, in some areas the species has begun to recover in recent years [26].

Due to the severe declines experienced by *R. sierrae* and its high susceptibility to infection with *B. dendrobatidis*, several studies have explored the skin microbiome of this species and its interaction with *B. dendrobatidis*. *B. dendrobatidis* infection can significantly disrupt the skin microbiome of *R. sierrae* [27] and this disruption is associated with reduced bacterial richness and evenness [28]. Both host genetics and environmental microbes influence the diversity and structure of the skin microbiome [29], and bacterial richness may be a predictor of host population extirpation or persistence in *B. dendrobatidis* epizootics [30].

In this study, we examine longitudinal patterns in the skin microbiome of 19 individually marked *R. sierrae*. We analyze the relationship between temporal bacterial community variability and α-diversity, the relative abundance of dominant taxa, and the proportion of ASVs likely to inhibit the growth of *B. dendrobatidis*. To better understand the patterns of variation observed, we investigate the relationship between ASV relative abundance, volatility, and transience. We also examine other possible sources of temporal variation in the skin microbiome, such as seasonality, population, sex, size, and *B. dendrobatidis* infection intensity. Finally, based on the temporal patterns we describe, we propose a set of criteria to use when selecting candidate bacteria for bioaugmentation interventions, and we present a set of bacteria from this dataset that meet those criteria.

## Methods

### Study site

Samples were collected from two populations in the Desolation Wilderness, CA (“Pyramid Valley” and “Rivendell Pond”). The populations are separated by ~3 km, and they occupy elevations ranging from 2450 to 2542 m. Both populations are found in habitats consisting primarily of small ponds and lakes connected by streams with extensive areas of exposed granite and sparsely distributed conifer trees. A network of lakes and streams containing non‐native fish lies between the two populations, which makes the habitat unsuitable for *R. sierrae* [31], and the two populations are not directly connected by water.

### Sample collection

Samples were collected from two populations (“Pyramid Valley” and “Rivendell Pond”) in the Desolation Wilderness, CA, as previously described [28]. In brief, frogs and tadpoles were handled using fresh gloves and were rinsed with 50 mL of sterilized pond water before being swabbed over the entire skin surface for 30 s with Dacron swabs. To quantify *B. dendrobatidis* infection intensity (i.e., load), a second set of swabs were collected from each animal using standard methods [24]. Samples were stored in microcentrifuge tubes on ice or dry ice for transportation to the laboratory and then stored at −80 °C until processing.

Samples were collected during June–September of 2013 and 2014, the summer period of peak *R. sierrae* activity. As part of a separate study of frog population dynamics at these two sites, 877 adult frogs ﻿(≥40 mm snout-vent length) were marked with 8 mm passive integrated transponder (PIT) tags inserted under the dorsal skin. Long-term population studies of *R. sierrae* utilizing PIT tags inserted in the same manner [32] and extensive observations of *R. sierrae* marked with PIT tags in captivity (Jessie Bushell, San Francisco Zoo, personal communication) have documented no negative effects on frog health. Microbiome samples were collected, as described above, from all marked adult frogs in the first sample collection period (June 2013) and from all recaptured frogs in the second sample collection period (July 2013). During August–September 2013 and June–September 2014, microbiome samples were collected only from frogs that had been captured in both the June and July 2013 sampling periods. Based on these criteria, a total of 19 individuals were sampled 2–6 times during the June 2013–September 2014 period (Supplemental Table S1). We performed 16S rRNA gene amplicon sequencing on the samples collected during each capture event from all 19 individuals.

### *B. dendrobatidis* sample processing and quantification

Samples for *B. dendrobatidis* quantification were collected and processed as previously described [28]. Briefly, *R. sierrae* were stroked 30 times on the ventral side using sterile Dacron swabs. Swabs were air-dried and stored at 4 °C until processing. DNA was extracted using PrepMan Ultra [33] and *B. dendrobatidis* infection intensity was quantified using quantitative PCR (qPCR) [34]. Zoospore equivalent (ZE) scores were calculated by multiplying the raw qPCR output by 80, to account for the subsampling and dilution that occurs during DNA extraction [24, 25].

### Temporal variation definitions

Because several kinds of temporal variation are explored in this study, we have adhered to the following terminology to disambiguate between different types of variation:

1. Variability: Variation in bacterial communities through time, defined as the average UniFrac distance between samples collected from an individual frog.
2. Volatility: Variation in the relative abundance of a particular bacterial ASV through time, defined as the SD of all relative abundances of the ASV for an individual frog.
3. Transience: For an individual frog and ASV, the fraction of samples in which the ASV was not detected.

### Microbiome sample preparation and sequencing

Samples were processed as described previously [28]. Briefly, DNA was extracted using the PowerSoil DNA Isolation Kit and sequencing libraries were prepared following the protocol “16S Metagenomic Sequencing Library Preparation” (Illumina, Inc., San Diego, CA, USA). The hypervariable V3–V4 region of the bacterial 16S gene was PCR amplified using primers with overhang adapters (Forward: 5′TCGTCGGCAGCGTCAGATGTGTATAAGAGACAGCCTACGGGNGGCWGCAG-3′; Reverse: 5′GTCTCGTGGGCTCGGAGATGTGTATAAGAGACAGGACTACHVGGGTATCTAATCC-3′). PCR product for triplicate reactions was pooled and purified using solid-phase reversible immobilization beads and dual indices were attached to the purified amplicons in a second round of PCR using the Nextera XT Index kit (Illumina, Inc.). The resulting PCR product was purified and quantified using qPCR (KAPA Library Quantification Kit; KAPA Biosystems), and samples were diluted and pooled at equimolar concentrations. Sequencing was performed on an Illumina MiSeq using a MiSeq Reagent Kit v3 (600 cycles) (Illumina, Inc.).

### Bioinformatics

Base calling and demultiplexing were performed using MiSeq Reporter (Illumina, Inc.). Unless otherwise specified, all bioinformatic analyses were performed using QIIME2 [35]. Trimming, quality control, and feature table construction was performed with dada2 using default settings [36]. Reverse reads were trimmed at position 179, forward reads were trimmed at position 299, and forward and reverse primers were trimmed. Sequences were aligned using MAFFT [37] and a phylogenetic tree was generated using FastTree2 [38]. A naive Bayes feature classifier was trained on the Greengenes 13_8 database [39] using the q2-feature-classifier plugin [40], and taxonomy was assigned using the q2-classify-sklearn plugin [41]. Sequences were discarded if identified as mitochondria (e.g., host DNA), chloroplast (e.g., algal DNA), or unclassified at phylum level.

Samples were rarefied at an even sampling depth of 5365 sequences for diversity analyses [42], which resulted in the exclusion of one sample from the analysis (SE26, a tadpole from Rivendell Pond, sampled in June 2014). Richness was quantified using Faith’s phylogenetic diversity [43] and evenness was quantified using Pielou’s evenness [44]. Differences in community structure were quantified using weighted UniFrac distances, and differences in community membership were quantified using unweighted UniFrac distances [45–49].

Differential abundance testing was performed using ANCOM [50] after filtering features representing fewer than 0.005% of the overall sequences and those present in fewer than 10% of the samples [51]. For analyses of ASV volatility, the full, unrarefied dataset was filtered to remove ASVs that represented fewer than 0.25% total sequences, in order to reduce bias from inconsistent detection of rare ASVs, following Oh et al. [13]. Sequences were then rarefied to a depth of 2955 per sample (which resulted in 3 samples being excluded: SE86, from adult A17 in August 2013; SE84, from adult A17 in June 2013; and SE72, from Adult A13 in June 2013). To identify ASVs present in the Woodhams database of *B. dendrobatidis*-inhibitory bacteria [52], q2-vsearch [53] was used with a closed reference feature clustering strategy and a clustering threshold of 99%.

### Statistical analyses

Unless otherwise noted, all statistical tests were performed in R [54]. For correlation analyses, such as those testing the relationship between microbiome variability and average α-diversity, Pearson’s correlation was used when test assumptions were met, and Spearman’s correlation was used otherwise. In order to check the assumptions of Pearson’s correlations, Shapiro–Wilk’s tests were used to test for normality, and Bartlett tests and Breusch–Pagan tests were used to test for homoskedasticity. To test for the effects of categorical variables (e.g., month, sex, frog ID, etc.) on community structure and membership, ADONIS was used, with population included as a covariate where applicable. For pairwise community comparisons between sample collection months, permutational multivariate analysis of variance (PERMANOVA) tests with Benjamini–Hochberg correction were used (ADONIS and PERMANOVA analyses were conducted using QIIME2). To test for differences between sample categories given a continuous response variable, such as in comparisons of air temperature between sample collection months, analysis of variance (ANOVA) was used when test assumptions were met, and Kruskal–Wallis was used otherwise. In order to check the assumptions of ANOVA, Shapiro–Wilk tests were used to test for normality, and Bartlett tests and Figner–Killeen tests were used to test for homogeneity of variances. To examine the relationship between ASV relative abundance and ASV volatility (variation in the relative abundance of a particular bacterial ASV through time), we used generalized additive models. To examine the relationship between ASV relative abundance and ASV transience (the fraction of samples in which the ASV was not detected), we binned ASVs into three relative abundance categories (<0.1%, 0.1–1%, and >1%), following Oh et al. [13], in order to minimize the impacts of technical limitations such as sampling depth, sequencing depth, and amplification bias. We tested for differences in transience between categories using Kruskal–Wallis tests with Dunn’s post hoc comparisons.

For analyses involving bacterial relative abundances, data were arcsine transformed for variance stabilization and to better approximate normality [55, 56]. For analyses involving infection intensity, *B. dendrobatidis* ZE scores were log transformed. For transience analyses, individuals with fewer than three samples were omitted (A18 and A19). To check for any bias introduced by uneven sampling between individuals, the analyses represented by all figures were conducted both with the full dataset and after randomly subsampling to three samples per individual; however, only minor differences were detected between the results of statistical tests between the two datasets.

### Air temperature

Air temperature data for the Desolation Wilderness was retrieved from the California Data Exchange Center (https://cdec.water.ca.gov/cdecstations) using station EP5, which is located ~7–9 km from the study populations, at an elevation ~70–170 m lower than sampling locations. Average daily air temperature at station EP5 was averaged over the 7-day period that included the sample collection date and the preceding 6 days. These average air temperature data were used to infer likely changes in water temperature at the study locations; previous research has shown that air temperature is closely correlated with water temperature in Sierra Nevada lakes [57].

## Results

### Individual microbiome variation

**Fig. 1:**
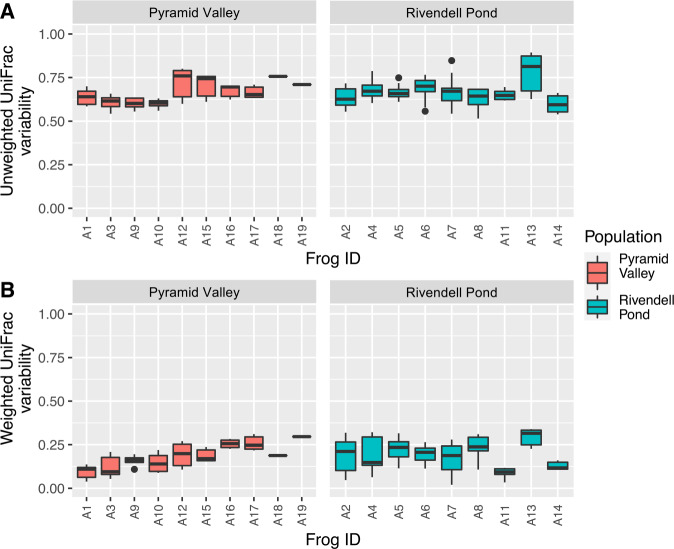
Individual microbiome variation. Community membership variability (**A**) and community structure variability (**B**). Each boxplot represents an individual frog.

Skin bacterial communities differed significantly between individual frogs, both in terms of community membership (*p* = 0.049, *R*^2^ = 0.237, *F* = 1.092, ADONIS) and community structure (*p* = 0.045, *R*^2^ = 0.270, *F* = 1.298, ADONIS). Community membership was much more variable over time than community structure in both populations: community membership variability ranged from 0.601 to 0.757 in the Pyramid Valley population and from 0.598 to 0.778 in the Rivendell Pond population (mean unweighted UniFrac distances; Fig. 1A), whereas community structure variability ranged from 0.099 to 0.296 in the Pyramid Valley population and from 0.089 to 0.294 in the Rivendell Pond population (mean weighted UniFrac distances; Fig. 1B). There was a significant negative correlation between the number of samples collected per individual and both community structure and membership variability in the Pyramid Valley Population, but not in the Rivendell Pond population (Supplemental Fig. S1).

### Microbiome variability and α-diversity

**Fig. 2:**
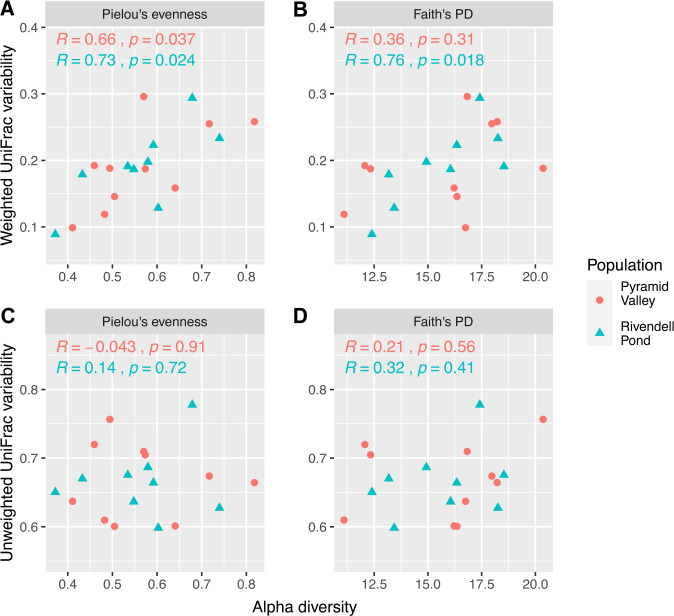
Microbiome variability and α-diversity. Scatterplots showing the relationship between α-diversity and community structure variability (**A**, **B**; weighted UniFrac distances) or community membership variability (**C**, **D**; unweighted UniFrac distances). Correlation coefficients and significance levels are Pearson’s correlations.

Among individual adult frogs, there was a significant positive correlation between average evenness and community structure variability in both populations (Fig. 2A, B). Individuals with low average evenness tended to have more temporal stability in their bacterial community structure compared to individuals with high average evenness. Richness was significantly correlated with community structure variability in the Rivendell Pond population, but not in the Pyramid Valley population. Community membership variability was not significantly correlated with evenness or richness in either population (Fig. 2C, D).

### Variability and bacterial order relative abundance

**Fig. 3:**
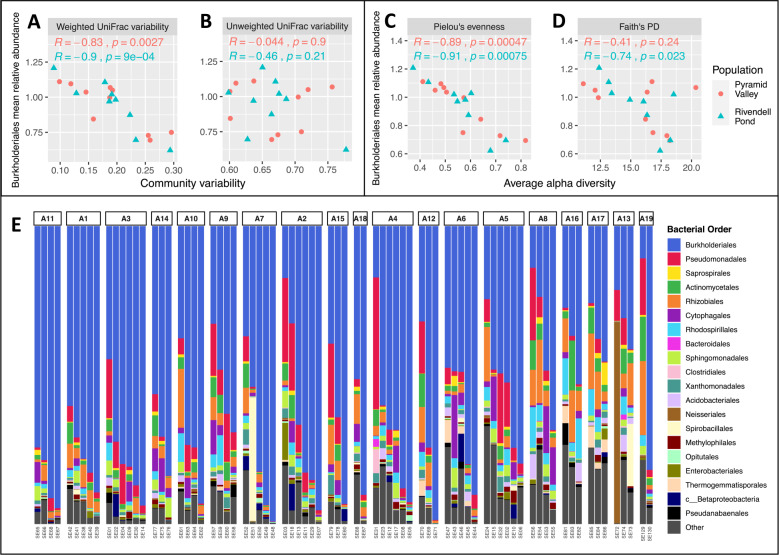
Variability and bacterial order relative abundance. **A**–**D** Scatterplots showing the relationship between an individual’s mean relative abundance of order Burkholderiales and the individual’s community variability (**A**, **B**) or average α-diversity (**C**, **D**). Correlation coefficients and significance levels are Pearson’s correlations. **E** Bar chart showing relative abundances of 20 most abundant bacterial orders. Each bar represents a sample and each group of bars represents an individual frog. Individuals are ordered by increasing community structure variability.

Community variability and average α-diversity were significantly correlated with the relative abundance of several bacterial orders. Order Burkholderiales, the most abundant bacterial order among adult frogs, was the only order that had a significant negative correlation with community variability and average α-diversity (i.e., the only order with significantly higher relative abundances among more stable and homogenous bacterial communities); all other bacterial orders had either a positive correlation with community variability and average α-diversity (i.e., higher relative abundances among more variable and diverse bacterial communities) or no significant correlation. There was a significant negative correlation between the average relative abundance of Burkholderiales and community structure variability, but not community membership variability, in both populations (Fig. 3A, B). There was also a significant negative correlation between the average relative abundance of Burkholderiales and average evenness, but there was a significant correlation with average richness only in the Rivendell Pond population (Fig. 3C, D). Orders that had a significant positive correlation with community variability in one population are shown in Supplemental Table S2 (no positive correlations were significant in both populations). Orders that had a significant positive correlation with α-variability in one population are shown in Supplemental Table S3 (again, no positive correlations were significant in both populations).

### ASV volatility and transience

**Fig. 4:**
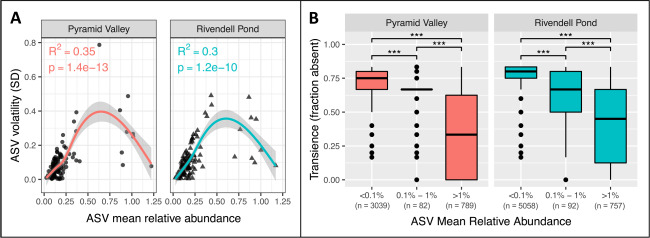
ASV volatility and transience. **A** Relationship between ASV mean relative abundance and volatility (SD) for each population, fitted with generalized additive models and 95% confidence intervals. ASVs representing <0.25% total relative abundance in the population were excluded from the figure and analyses. **B** ASV transience, defined as the fraction of samples in an individual time series in which the ASV was not detected, binned in three mean relative abundance categories. Asterisks represent statistical significance (*<0.05, **<0.01, and ***<0.001) for Kruskal–Wallis tests with Dunn’s post hoc comparisons. Individuals with fewer than three samples were excluded from the figure and analyses.

To investigate patterns of variation among bacterial taxa that could drive the observed differences in community structure variability, we examined the relationship between ASV relative abundance, volatility, and transience. We found that ASVs with intermediate-level relative abundances experienced the most volatility over time; those with lower or higher relative abundances tended to be more consistent (Fig. 4A and Supplemental Fig. S2). ASV volatility was not significantly correlated with number of samples per individual in either population (Supplemental Fig. S3). Transience differed significantly between relative abundance bins: ASVs with low relative abundance were the most transient, and ASVs with high relative abundance were the most persistent (Fig. 4B). ASV transience had a weak but significant positive correlation with the number of samples per individual in the Rivendell Pond population, but no significant correlation in the Pyramid Valley population (Supplemental Fig. S4).

### Likely *B. dendrobatidis*-inhibitory bacteria

**Fig. 5:**
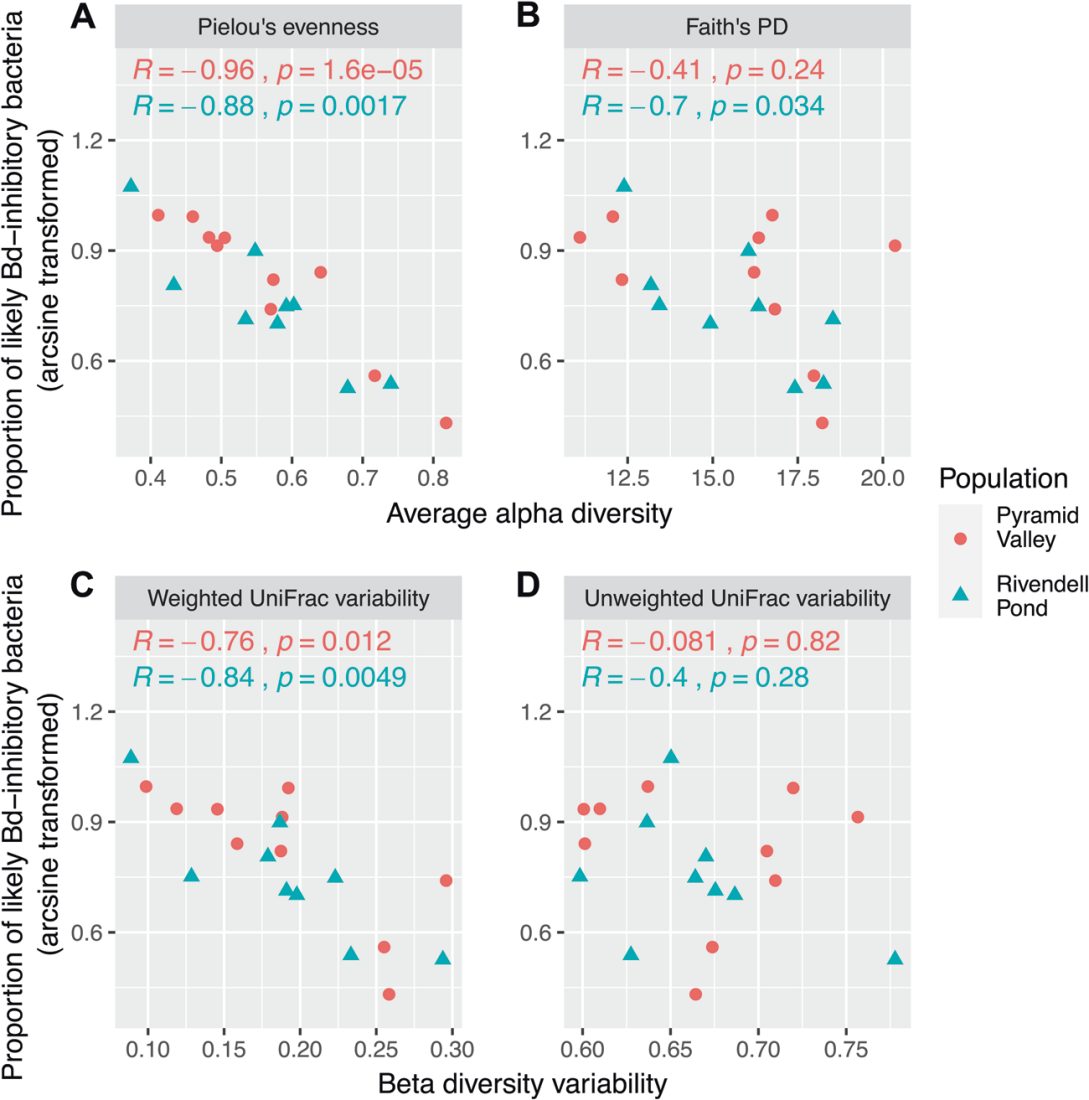
Likely B. dendrobatidis-inhibitory bacteria. Scatterplots showing the relationship between an individual’s average proportion of likely *B. dendrobatidis* (*Bd*)-inhibitory bacteria and the individual’s average α-diversity (**A**, **B**) or community variability (**C**, **D**). Correlation coefficients and significance levels are Pearson’s correlations.

To examine possible functional changes correlated with the longitudinal patterns described in the previous sections, we compared our 16S sequence dataset to the database of *B. dendrobatidis*-inhibitory bacteria published in Woodhams et al. [52], and we calculated the percentage of sequences within each sample that clustered at a threshold of 99% identity with isolates shown to have a significant inhibitory effect on the growth of *B. dendrobatidis*. We found that, among individual frogs, average evenness had a significant negative correlation with the average proportion of likely *B. dendrobatidis*-inhibitory bacteria. Richness had a significant negative correlation with the average proportion of likely *B. dendrobatidis*-inhibitory bacteria in the Rivendell population, but not the Pyramid population (Fig. 5A, B). Variability in community structure likewise had a significant negative correlation with the proportion of likely *B. dendrobatidis*-inhibitory bacteria, but there was no significant correlation with variability in community membership (Fig. 5C, D). The proportion of likely *B. dendrobatidis*-inhibitory bacteria was not significantly correlated with any other frog or sample attributes examined in either population, including month, year, frog sex (all *p* > 0.05; ANOVA), temperature, frog length, and *B. dendrobatidis* infection intensity (all *p* > 0.05; Spearman’s correlation).

### Seasonal variation

**Fig. 6:**
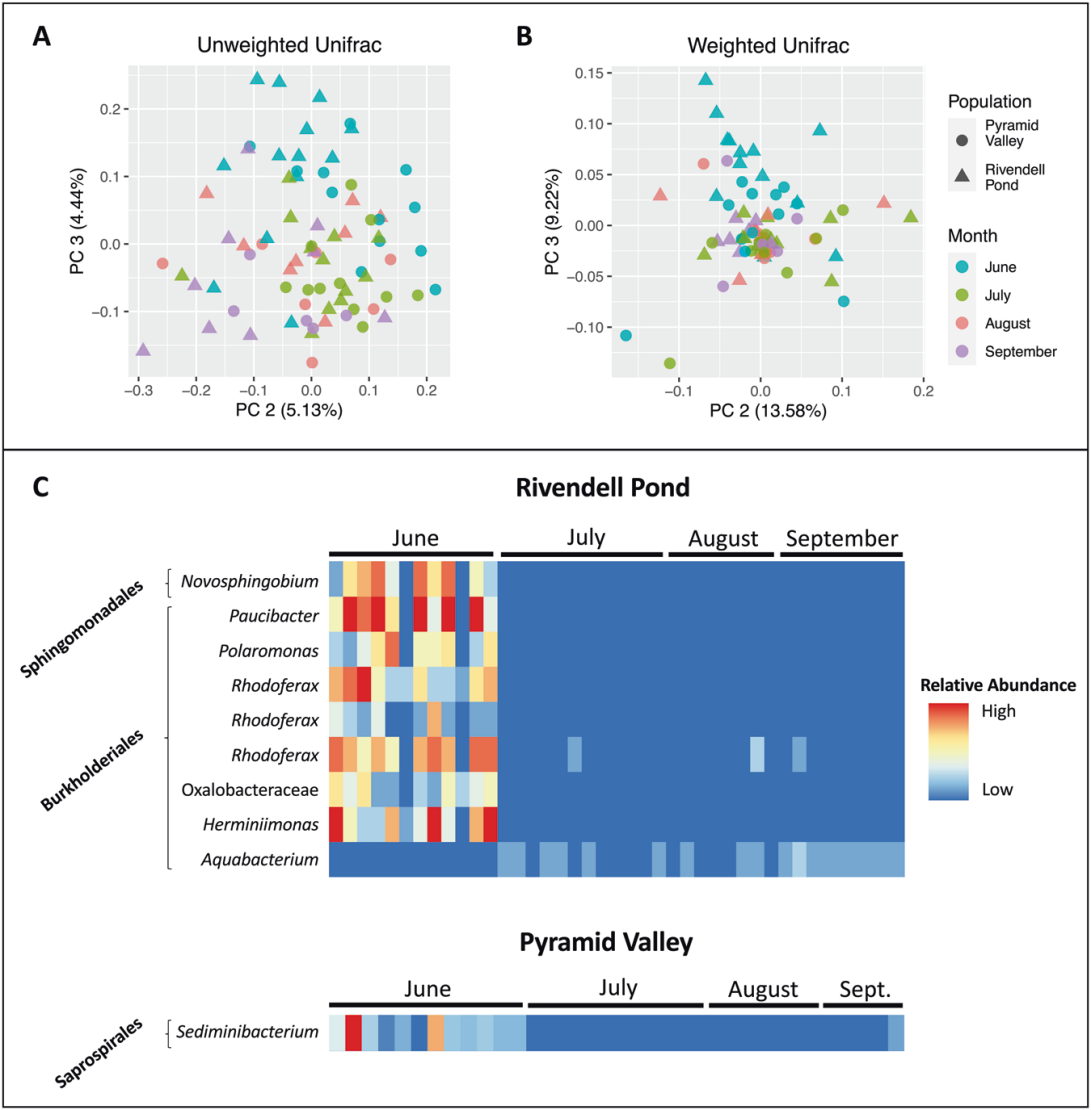
Seasonal variation. **A**, **B** Principle coordinates analysis (PCoA) plots, using unweighted and weighted UniFrac distances. PC2 and PC3 are shown, as these axes were most strongly associated with seasonal differences between samples. **C** Relative abundances of bacterial orders identified as most strongly correlated with differences between months using ANCOM. Each column represents a sample and each row represents a bacterial ASV.

In order to identify other factors that may be associated with temporal changes in the skin microbiome, we analyzed seasonal patterns and explored the effects of frog attributes such as sex, size, population, and *B. dendrobatidis* infection intensity. The bacterial communities of frogs differed significantly based on sample collection month in both populations, both in terms of community membership (Pyramid: *p* = 0.008, *R*^2^ = 0.113, *F* = 1.318; Rivendell: *p* = 0.003, *R*^2^ = 0.109, *F* = 1.543; ADONIS) and community structure (Pyramid: *p* = 0.049, *R*^2^ = 0.141, *F* = 1.690; Rivendell: *p* = 0.008, *R*^2^ = 0.134, *F* = 1.963; ADONIS; Fig. 6). Samples collected in June were most consistently distinct from samples collected in other months, in both populations (pairwise PERMANOVA tests with Benjamini–Hochberg correction; Table 1). Richness and evenness did not differ significantly between months in either population (all *p* > 0.05; Kruskal–Wallis tests).

**Table 1:** Pairwise bacterial community comparisons between sample collection months.

Community membership (unweighted UniFrac)									
**Pyramid Valley**					**Rivendell Pond**				
June	−				June	−			
July	0.068	−			July	0.018*	−		
August	0.030*	0.418	−		August	0.021*	0.506	−	
September	0.030*	0.050*	0.641	−	September	0.036*	0.036*	0.236	−
	June	July	August	September		June	July	August	September

Community structure (weighted UniFrac)									
**Pyramid Valley**					**Rivendell Pond**				
June	−				June	–			
July	0.228	−			July	0.015*	–		
August	0.042*	0.228	−		August	0.066	0.488	−	
September	0.228	0.623	0.623	−	September	0.015*	0.219	0.540	−
	June	July	August	September		June	July	August	September

Cell contents are *p*-values for pairwise PERMANOVA tests with Benjamini–Hochberg correction. Asterisks represent statistical significance (*<0.05, **<0.01, and ***<0.001).

To explore possible causes for the seasonal patterns observed, we examined the relationship between bacterial communities and average air temperature during the week preceding sample collection. All months differed significantly from one another in temperature (all *p* < 0.05; ANOVA with Tukey’s post hoc comparisons), with June having the most different (lowest) temperature. Differences in community structure were significantly correlated with air temperature in the Pyramid Valley population (*p* = 0.028, *ρ* = 0.096) but not the Rivendell Pond population (*p* = 0.225, *ρ* = 0.063, Mantel’s tests with Spearman’s correlations), whereas differences in community membership were not significantly correlated with air temperature in either population (Pyramid: *p* = 0.953, *ρ* = −0.002; Rivendell: *p* = 0.496, *ρ* = 0.030, Mantel’s tests with Spearman’s correlations). Air temperature was not significantly correlated with richness or evenness in either population (all *p* > 0.05; Spearman’s correlations).

### Population and frog attributes

There was a significant difference in the skin microbiome of frogs between the two populations (Supplemental Fig. S5). Community membership differed between the two populations (*p* = 0.012, *R*^2^ = 0.021, *F* = 1.605, ADONIS), but community structure did not (*p* = 0.155, *R*^2^ = 0.019, *F* = 1.435, ADONIS). ANCOM analysis suggests that the difference between the populations is driven primarily by differential abundances in the orders Enterobacteriales and Rhizobiales. There was also no significant difference in richness (*p* = 0.967, *H* = 0.002, Kruskal–Wallis) or evenness (*p* = 0.902, *H* = 0.015, Kruskal–Wallis) between the populations. There was no significant difference between frog sexes based on any of the α-diversity (all *p* > 0.05, Kruskal–Wallis) or β-diversity (all *p* > 0.05, ADONIS) metrics examined in either population. Similarly, there was no significant correlation between snout-vent length or *B. dendrobatidis* infection intensity with any of the α-diversity (all *p* > 0.05, Spearman’s correlation) or β-diversity (all *p* > 0.05, Mantel test) metrics examined in either population. For context, *B. dendrobatidis* infection levels were generally low among frogs included in this study: the maximum infection intensity was 552 ZE and the mean infection intensity was 37.5 ZE (Supplemental Table S1; mortality is typically observed only in post-metamorphic *R. sierrae* with infection intensities exceeding ~10,000 ZE).

## Discussion

### Temporal variation in the *R. sierrae* skin microbiome

In this study, we investigated (1) the relationship between community variability, diversity, and predicted anti-*B. dendrobatidis* function in the skin microbiome of *R. sierrae*, (2) the relationship between ASV relative abundance, volatility, and transience, and (3) how the microbiome is impacted by seasonality, population, and other individual characteristics. We found that community structure was more stable through time than community membership among all frogs included in the study. Community structure variability, but not community membership variability, was positively correlated with evenness and negatively correlated with the relative abundance of the dominant bacterial order Burkholderiales. In analyses of individual bacterial ASVs, ASV volatility was highest in intermediate-abundance ASVs, whereas ASV transience was highest in low-abundance ASVs. These patterns of ASV variation may explain the negative correlation observed between community structure variability and evenness, as the microbiome of low-evenness individuals is typically dominated by a small number of high-abundance ASVs with low volatility and low transience (more stable), whereas the microbiome of high-evenness individuals is typically dominated by a higher number of intermediate-abundance ASVs with high volatility and intermediate transience (less stable). In an exploration of potential microbial community function, we found that the proportion of likely *B. dendrobatidis*-inhibitory bacteria was negatively correlated with α-diversity and community structure variability. Finally, we found that community membership differed seasonally in both populations, with especially strong shifts observed among samples collected in June, when frogs are mating. Microbial communities differed significantly between the two study populations, but no significant correlations were found based on sex, size, or *B. dendrobatidis* infection intensity.

### Community structure variation vs. community membership variation

Just as we found that community structure was more stable through time than community membership, the first longitudinal study of the skin microbiome in humans found that community structure was more stable than community membership across each of 19 skin sites examined [58]. The frequent contact of the skin with external substrates may help to explain the high turnover observed in community membership in both systems. It is possible that measures of community membership variability may be biased by inconsistent detection of low relative abundance ASVs, whereas measures of community structure variability are more strongly influenced by fluctuations in the relative abundance of more dominant ASVs; thus, some of the differences we observed between community membership and community structure variability may be an artifact of sequencing methods. Community variability correlations in the Pyramid Valley population may also be influenced by unequal numbers of samples collected per individual. Interestingly, although we found that community structure variability was positively correlated with α-diversity and negatively correlated with the relative abundance of Burkholderiales, we found no consistently significant longitudinal correlations with community membership variability. This suggests that all frogs in our study populations experienced a high level of ASV turnover, especially among rare ASVs, even though some individuals experienced significantly more changes in the relative abundances of dominant taxa. Thus, the “signature” of an individual frog’s microbiome may be better represented by community structure than by community membership.

### Comparing longitudinal patterns in the skin microbiome of amphibians and humans

Many of the results of our longitudinal analyses were strikingly similar to those described in the most comprehensive study to date exploring temporal dynamics in the human skin microbiome [13]. Just as we found that variability in bacterial community structure was positively correlated with evenness, Oh et al. [13] found that variability in microbial community structure was positively correlated with Shannon diversity (an index that incorporates both evenness and richness). In both studies, microbial taxa with intermediate levels of relative abundance experienced the greatest volatility, and microbial taxa with lower relative abundances experienced the most transience. Analyses of ASV transience may be biased towards finding higher transience among low relative-abundance ASVs because of reduced detectability among such rare ASVs due to technical limitations, such as sampling depth, sequencing depth, and amplification bias; however, both our study and the Oh et al. [13] study attempted to limit the impact of these biases in our analyses by binning ASVs into broad relative abundance categories rather than treating relative abundance as a continuous variable. It may be that the volatility and transience of component ASVs is a main driver of the variability of microbial communities: low-evenness communities are typically dominated by a small number of high-abundance ASVs, which are likely to have low volatility and transience; thus, they experience low temporal variability. Alternatively, high-evenness communities are typically dominated by a larger number of intermediate-abundance ASVs, which are likely to have high volatility and intermediate transience; thus, they experience higher temporal variability. The congruence of findings between *R. sierrae* and humans is especially interesting given that the human skin microbiome experiences a high degree of regular disturbance, e.g., through bathing with soaps, etc., whereas the animals in our study live in wild populations without such regular disturbances. As our study is the first to explore longitudinal patterns in the skin microbiome of amphibians in wild populations (and, to our knowledge, the first to explore longitudinal patterns in the skin microbiome of any animal host in wild populations), it remains unknown whether similar host–microbiome relationships are found in other amphibians, and in other organisms more generally. We suggest that extending our approach to other taxa should be a high priority for future studies.

### *B. dendrobatidis* and the microbiome

In this study, we found that *B. dendrobatidis* infection intensity was not significantly correlated with community structure, membership, or diversity. This finding may seem somewhat surprising, given that our previous analysis [28] and several other studies [27, 59] found that infection with *B. dendrobatidis* causes or is associated with changes in the microbiome. This seeming contradiction is likely explained by the enzootic disease dynamics currently at play in the specific populations we studied: recently metamorphosed juveniles experience high *B. dendrobatidis* infection intensities (>10,000 ZE) and associated mortality [24, 60], whereas surviving adults are nearly all infected with *B. dendrobatidis* at low, sublethal levels [28]. Meanwhile, studies that have found significant correlations between *B. dendrobatidis* infection and microbial community structure [27, 59] focused on populations experiencing epizootic outbreaks of *B. dendrobatidis*, with widespread, severe *B. dendrobatidis* infection intensities. In our previous analysis, which included both adult and juvenile frogs, we found that significant correlations between *B. dendrobatidis* and the microbiome only occur in frogs with high *B. dendrobatidis* infection levels (>1000 ZE). All frogs included in the current study were adults, with infection intensities that were consistently far lower than this 1000 ZE threshold (Supplemental Table S1). Thus, the lack of high infection intensities among frogs included in this study likely explains the lack of significant interactions detected between *B. dendrobatidis* and the microbiome.

### Seasonality

We detected significant differences between sample collection months, similar to other recent studies [16, 18, 19]. Differences were significant in both community structure and community membership (differences in community structure were near significant in the Pyramid Valley population), but ANCOM shows that different ASVs were most strongly correlated with seasonal differences in the two populations. In both populations, samples collected in June were consistently the most different from samples collected in other months, and this pattern may be explained by changes in host habitat use or reproductive state. Frogs in our study populations typically overwinter in ponds or lakes (typically October–May) and they breed in their overwintering habitat as soon as the water body thaws, usually in June. They spend much of the rest of the summer active season foraging in nearby streams, smaller ponds, and fringing aquatic habitat. Thus, changes in habitat use or reproductive state (in addition to environmental factors, such as water temperature) may explain the differences observed between samples collected in June and those collected in other months.

### Likely *B. dendrobatidis*-inhibitory bacteria

We found that individuals with lower community structure variability and lower α-diversity had a significantly higher proportion of likely *B. dendrobatidis*-inhibitory bacteria than those with higher community structure variability or diversity. This is likely due to the high relative abundance in such individuals of ASVs in order Burkholderiales that have been found to inhibit the growth of *B. dendrobatidis* in laboratory trials. It should also be noted that the proportion of bacteria not known to be inhibitory includes some species with a documented nonsignificant or enhancing effect, but mostly bacteria whose interactions with *B. dendrobatidis* have not been evaluated. Thus, it is possible that these patterns would change or disappear if a higher proportion of the ASVs we detected had been evaluated for *B. dendrobatidis*-inhibitory properties. In a seemingly contradictory finding, we found in a previous study that the relative abundance of Burkholderiales was significantly higher in highly infected *R. sierrae* than those with lower infection levels [28], and Jani and Briggs [27] also found that several taxa in order Burkholderiales have a significant positive correlation with *B. dendrobatidis* infection intensity. In our previous paper, we hypothesized that the increase in the relative abundance of Burkholderiales, and associated decrease in α-diversity, could be a form of dysbiosis, restricting the range of ecological functions performed by the skin microbiome and potentially leading to negative health outcomes for hosts, or that it could increase community invasibility. However, the findings described here suggest that it is also possible that the changes that *B. dendrobatidis* causes in the skin microbiome, such as those documented by Jani and Briggs [27], actually bolster the host’s defenses, by increasing the proportion of bacteria that inhibit the growth of *B. dendrobatidis*. Gaining a more complete understanding of the relationship between the microbiome and *B. dendrobatidis* infection will require a more thorough description of the *B. dendrobatidis*-inhibitory properties of the bacteria that compose the microbiome.

### Recommendations for bioaugmentation interventions

Although thousands of bacterial isolates have been cultured and tested for *B. dendrobatidis*-inhibitory properties [52], the role of proposed candidates for probiotic treatments within the context of the temporally dynamic skin microbiome has rarely been assessed. As the success of bioaugmentation trials in the wild has often been hampered by diminishing abundance and increasing transience of the probiotic target [7, 10, 11, and Vredenburg, unpublished data], we suggest that longitudinal patterns in the microbiome, specifically ASV volatility and transience, should be used to inform future bioaugmentation experiments. Here we found that ASVs with low relative abundance are more likely to be transient and are therefore perhaps less likely to successfully persist through time in a bioaugmentation intervention. ASVs with intermediate relative abundance are more likely to persist than low-abundance ASVs, but they experience the highest degree of volatility. Thus, probiotics in this category may not provide a consistent defense against *B. dendrobatidis*, e.g., through inconsistent production of antifungal metabolites. Assuming that the patterns of microbiome variability, transience, and volatility described in this study for *R. sierrae* are relevant to microbiomes of amphibians more generally, we propose that ASVs with high relative abundance in at least some individuals are the best candidates for bioaugmentation interventions, as bacteria with these characteristics have the demonstrated ability to take a dominant role within the skin bacterial community of the target amphibian. It is possible that inoculation of amphibians with high levels of such bacterial strains could shift the skin microbiome to an alternative stable state—one in which the target bacterial strain is shifted to a dominant role in the microbial community with low enough levels of transience and volatility to provide reliable protection from *B. dendrobatidis* in the long term. Below, we present a table of six ASVs with high mean relative abundance, low volatility, low transience, and which have either been shown to significantly inhibit the growth of *B. dendrobatidis*, or whose interactions with *B. dendrobatidis* have not yet been assessed. We propose that these ASVs are likely good candidates for the development of probiotic treatments (Table 2) and we suggest that efforts to develop bioaugmentation interventions in other systems should examine the temporal stability of candidate strains before attempting interventions in the wild.

**Table 2:** Proposed candidates for probiotic treatments.

Taxonomy	Mean	Volatility (SD)	Transience (fraction absent)	Inhibitory?	Woodhams et al. [52] ID
Cytophagales					
* Flectobacillus sp*	31%	14%	0/3	Not assessed	−
Pseudomonadales					
* Pseudomonas sp1*	25%	7%	3/6	Inhibitory	Ranamuscosa-inhibitory_83
* Pseudomonas sp2*	22%	4%	2/4	Inhibitory	Ranamuscosa-inhibitory_6
Rhizobiales					
* Methylobacterium adhaesivum*	37%	11%	0/3	Not assessed	−
* Methylobacterium sp1*	22%	6%	0/3	Not assessed	−
* Methylobacterium sp2*	25%	10%	0/3	Not assessed	−

ASVs included have high mean relative abundance, low volatility, and transience, and are either proven to inhibit the growth of *B. dendrobatidis* or not yet assessed.

## Supplementary information

Supplementary information
