# Supplementary material for: Longitudinal patterns in the skin microbiome of wild, individually marked frogs from the Sierra Nevada, California
Source: ISME Commun. 2021 Sep 1;1:45. doi: 10.1038/s43705-021-00047-7 (PMC9723788; doi:10.1038/s43705-021-00047-7)
Supplement: Supplementary file 1 — Supplementary information [file 43705_2021_47_MOESM1_ESM.pdf]

Frog ID	Population	Sex	# Samples	Mean SVL	Mean Weight	Mean ZE
A1	Pyramid Valley	Male	5	52.2	18.1	46.4
A2	Rivendell Pond	Male	6	45.4	11.1	50.6
A3	Pyramid Valley	Male	6	49.9	13.5	44.0
A4	Rivendell Pond	Female	6	46.7	12.7	28.7
A5	Rivendell Pond	Female	6	48.6	13.5	10.7
A6	Rivendell Pond	Female	5	50.3	14.5	26.3
A7	Rivendell Pond	Female	5	44.0	12.3	14.4
A8	Rivendell Pond	Female	4	48.1	13.5	8.4
A9	Pyramid Valley	Male	4	53.2	15.3	45.5
A10	Pyramid Valley	Female	4	69.4	43.8	32.1
A11	Rivendell Pond	Female	4	48.9	13.0	3.4
A12	Pyramid Valley	Male	3	53.3	17.2	247.9
A13	Rivendell Pond	Male	3	48.8	13.8	0.0
A14	Rivendell Pond	Female	3	52.3	14.2	29.5
A15	Pyramid Valley	Male	3	45.9	10.7	5.5
A16	Pyramid Valley	Male	3	54.6	18.3	25.5
A17	Pyramid Valley	Male	3	46.3	12.0	26.4
A18	Pyramid Valley	Male	2	47.2	13.0	0.0
A19	Pyramid Valley	Male	2	54.1	15.8	140.8

Supplemental Table S1. Adult frogs included in the study.

Pyramid Valley

	Weighted UniFrac		*Unweighted UniFrac*	
**Order**	**R**	**p**	**R**	**p**
Actinomycetales	0.70	0.025*	0.10	0.803
Rhizobiales	0.83	0.003*	0.60	0.067*

Rivendell Pond

	Weighted UniFrac		*Unweighted UniFrac*	
**Order**	**R**	**p**	**R**	**p**
Acidobacteriales	0.87	0.002*	0.33	0.381
Armatimonadales	0.83	0.005*	0.22	0.576
Caulobacterales	0.70	0.036*	0.39	0.308
Gemmatales	0.73	0.025*	0.14	0.732
Ktedonobacterales	0.63	0.067	0.77	0.016*
Myxococcales	0.78	0.013*	0.48	0.187
Pseudanabaenales	0.73	0.025*	0.48	0.187
Rhodospirillales	0.93	0.000**	0.17	0.668
Solibacterales	0.90	0.001**	0.50	0.170
Sphingomonadales	0.00	1.000	0.77	0.016*
Spirobacillales	0.71	0.030*	0.62	0.077

Supplemental Table S2. Orders with a significant positive correlation with community variability in one population. Correlation coefficients and significance levels are for Spearman correlations; asterisks represent statistical significance (*<0.05; **<0.001; ***<0.0001).

Pyramid Valley

	Pielou's evenness		*Faith's PD*	
**Order**	**R**	**p**	**R**	**p**
Armatimonadales	0.60	0.067*	0.63	0.048*
Gemmatales	0.61	0.060*	0.71	0.022*
Rhizobiales	0.53	0.117	0.73	0.016*
Rhodospirillales	0.66	0.038*	0.26	0.446
Solibacterales	0.69	0.029*	0.70	0.025*
Synechococcales	0.54	0.108	0.77	0.038*

Rivendell Pond

	Pielou's evenness		*Faith's PD*	
**Order**	**R**	**p**	**R**	**p**
Acidobacteriales	0.77	0.016*	0.73	0.025*
Actinomycetales	0.85	0.004*	0.63	0.067*
Armatimonadales	0.48	0.187	0.78	0.013*
Caulobacterales	0.30	0.433	0.83	0.005*
Gemmatales	0.80	0.010*	0.44	0.244
Myxococcales	0.33	0.381	0.88	0.002*
Pedosphaerales	0.33	0.381	0.75	0.020*
Pseudomonadales	0.41	0.265	0.70	0.036*
Rhizobiales	0.71	0.030*	0.14	0.732
Rhodospirillales	0.78	0.013*	0.78	0.013*
Saprospirales	0.78	0.013*	0.50	0.17
Solibacterales	0.53	0.139	0.71	0.030*
Xanthomonadales	0.17	0.637	0.69	0.042*

Supplemental Table S3. Orders with a significant positive correlation with alpha diversity. Correlation coefficients and significance levels are for Spearman correlations; asterisks represent statistical significance (*<0.05; **<0.001; ***<0.0001).

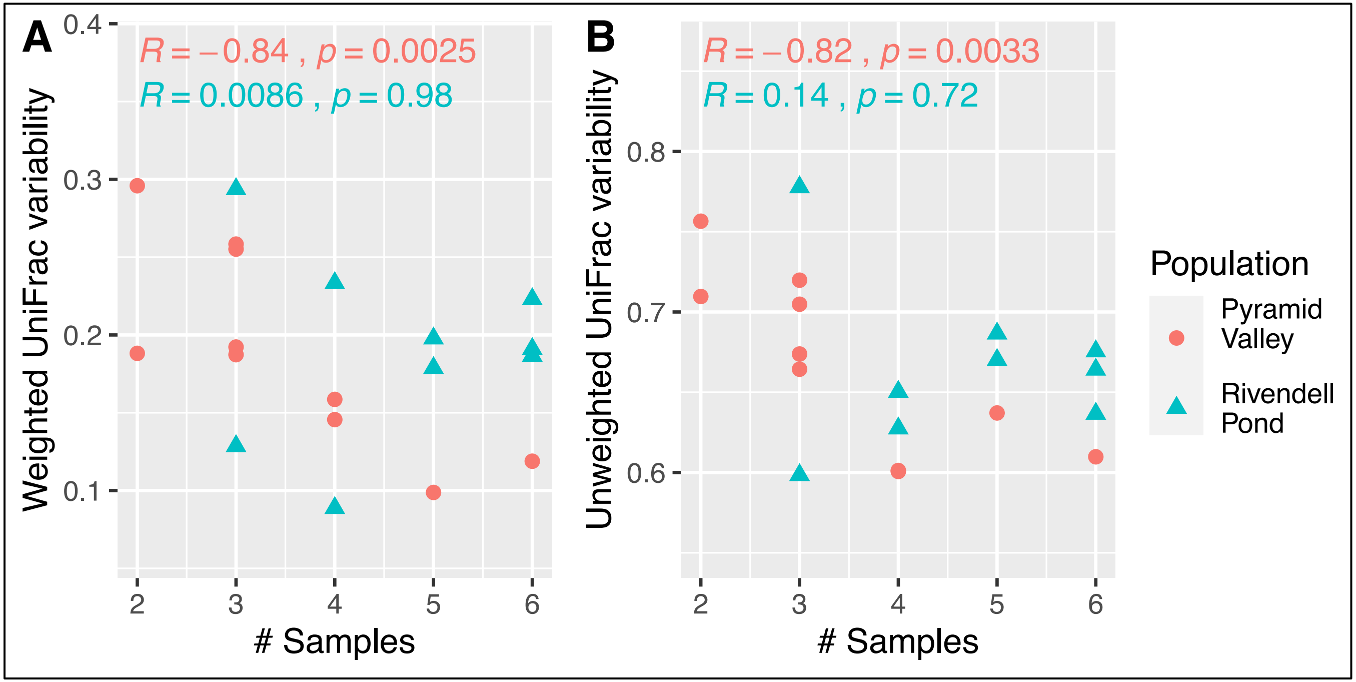

Supplemental Figure S1. Scatterplots showing the relationship between the number of samples collected per individual frog and community structure variability (A; weighted UniFrac distances) or community membership variability (B; unweighted UniFrac distances). Correlation coefficients and significance levels are Spearman correlations.

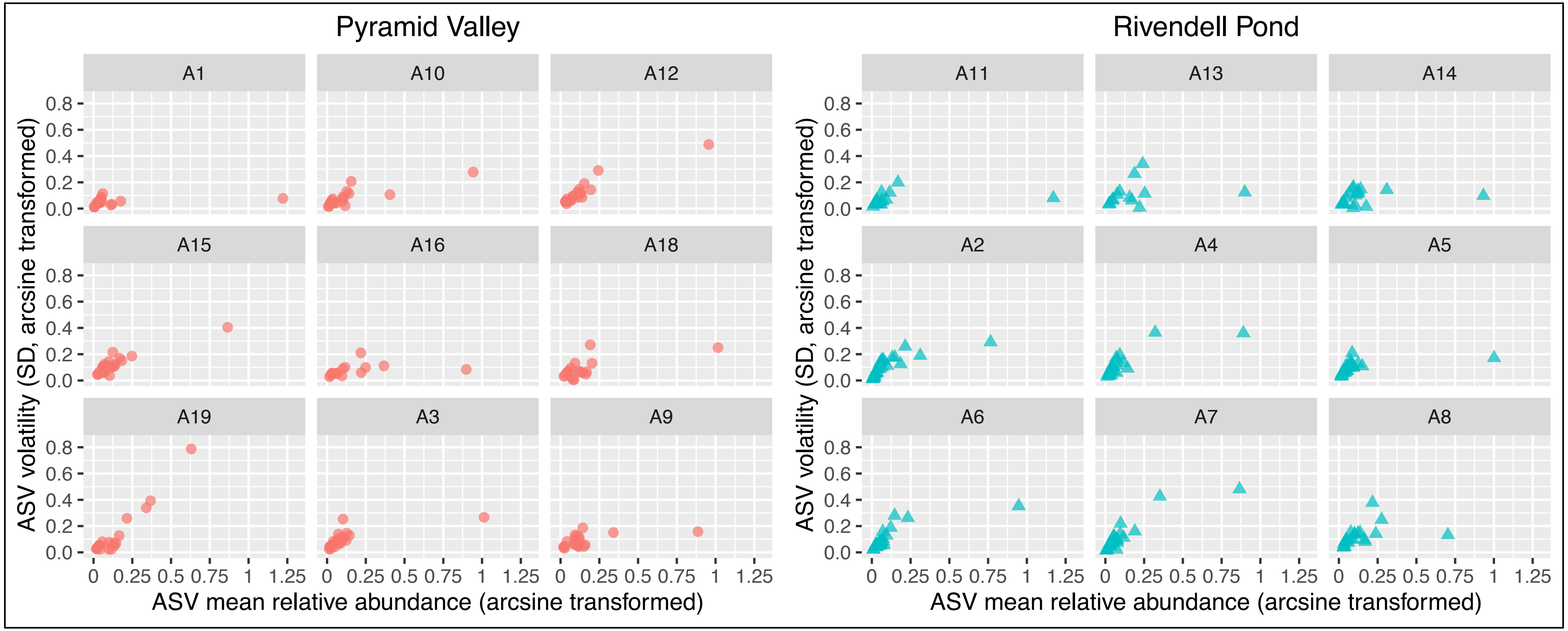

Supplemental Figure S2. Scatterplots showing ASV volatility (SD) vs. ASV mean relative abundance for each individual frog.

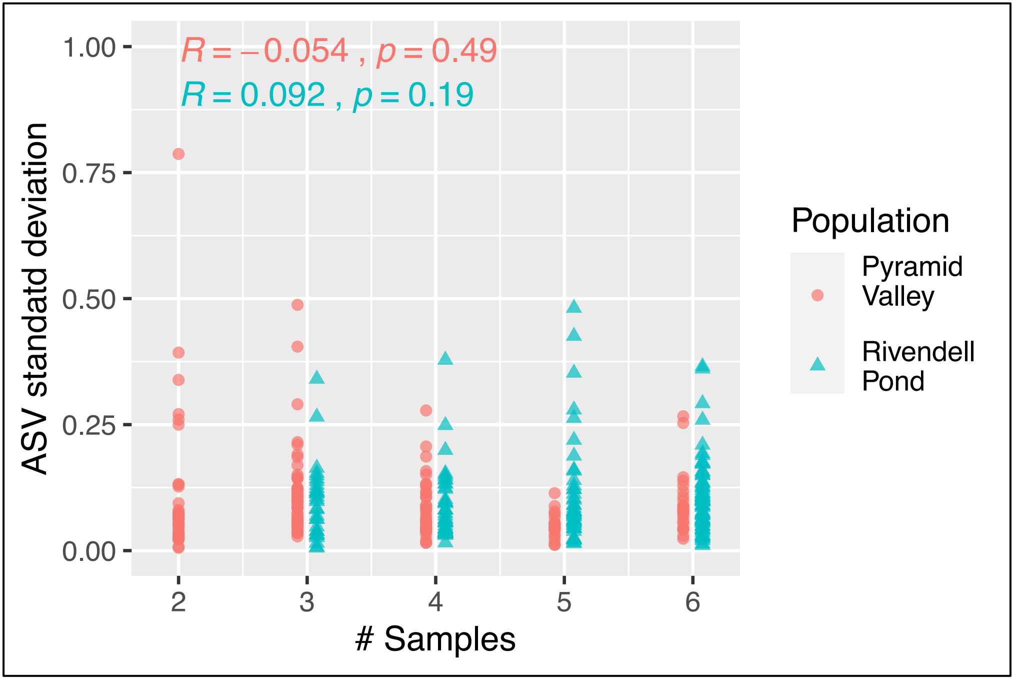

Supplemental Figure S3. Scatterplots showing the relationship between the number of samples collected per individual frog and ASV volatility. Correlation coefficients and significance levels are Spearman correlations.

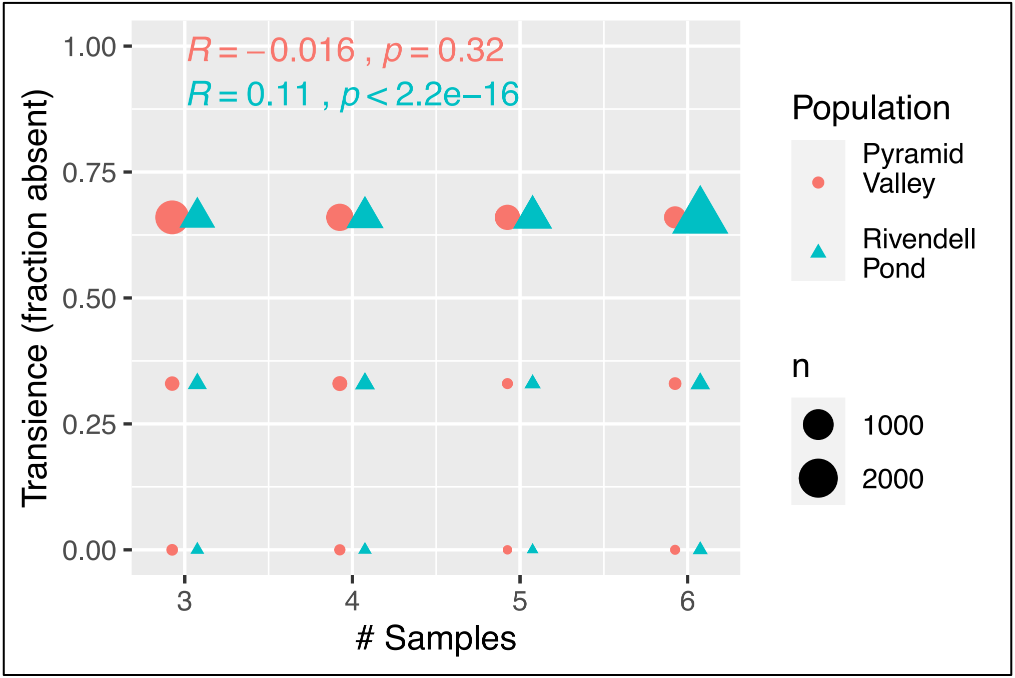

Supplemental Figure S4. Scatterplots showing the relationship between the number of samples collected per individual frog and ASV transience, after rounding to the nearest 33% (to correct for the increasing upper transience limit with increasing number of samples collected per individual). Correlation coefficients and significance levels are Spearman correlations.

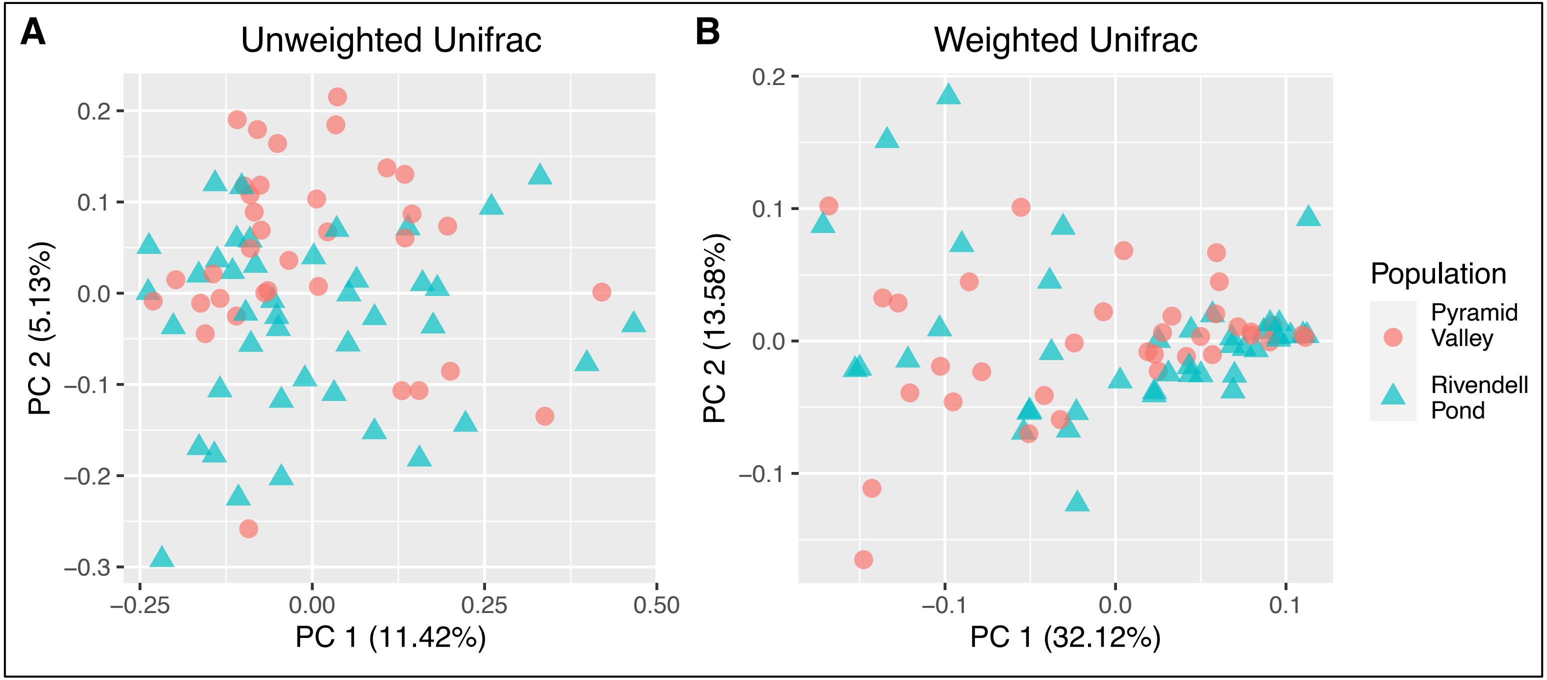

Supplemental Figure S5. Principle coordinates analysis (PCoA) plots, using unweighted UniFrac distances (A) and weighted UniFrac distances (B).
